# Determinant of Covariance Matrix Model Coupled with AdaBoost Classification Algorithm for EEG Seizure Detection

**DOI:** 10.3390/diagnostics12010074

**Published:** 2021-12-29

**Authors:** Hanan Al-Hadeethi, Shahab Abdulla, Mohammed Diykh, Jonathan H. Green

**Affiliations:** 1 School of Sciences, University of Southern Queensland, Toowoomba, QLD 4300, Australia; Hananalihamood.alhadeethi@usq.edu.au; 2 USQ College, University of Southern Queensland, Toowoomba, QLD 4300, Australia; 3 College of Education for Pure Science, University of Thi-Qar, Nasiriyah 64001, Iraq; 4 Information and Communication Technology Research Group, Scientific Research Centre, Al-Ayen University, Nasiriyah 64001, Iraq; 5 Faculty of the Humanities, University of the Free State, Bloemfontein 9301, South Africa

**Keywords:** Electroencephalography, Cov–Det, epileptic AB_BP_NN, KST, MWUT

## Abstract

Experts usually inspect electroencephalogram (EEG) recordings page-by-page in order to identify epileptic seizures, which leads to heavy workloads and is time consuming. However, the efficient extraction and effective selection of informative EEG features is crucial in assisting clinicians to diagnose epilepsy accurately. In this paper, a determinant of covariance matrix (Cov–Det) model is suggested for reducing EEG dimensionality. First, EEG signals are segmented into intervals using a sliding window technique. Then, Cov–Det is applied to each interval. To construct a features vector, a set of statistical features are extracted from each interval. To eliminate redundant features, the Kolmogorov–Smirnov (KST) and Mann–Whitney U (MWUT) tests are integrated, the extracted features ranked based on KST and MWUT metrics, and arithmetic operators are adopted to construe the most pertinent classified features for each pair in the EEG signal group. The selected features are then fed into the proposed AdaBoost Back-Propagation neural network (AB_BP_NN) to effectively classify EEG signals into seizure and free seizure segments. Finally, the AB_BP_NN is compared with several classical machine learning techniques; the results demonstrate that the proposed mode of AB_BP_NN provides insignificant false positive rates, simpler design, and robustness in classifying epileptic signals. Two datasets, the Bern–Barcelona and Bonn datasets, are used for performance evaluation. The proposed technique achieved an average accuracy of 100% and 98.86%, respectively, for the Bern–Barcelona and Bonn datasets, which is considered a noteworthy improvement compared to the current state-of-the-art methods.

## 1. Introduction

Epilepsy is a brain disorder characterized by abnormal discharge of neurons and by seizures that can lead to cognitive, psychological and social consequences [1,2,3,4,5,6,7]. Based on the latest report on epilepsy released by the World Health Organization (WHO), more than 50 million people worldwide have this disease [8,9]. The number of people with epilepsy is expected to increase further thanks to increasing life expectancy and the higher ratio of people surviving birth trauma, traumatic brain injury, infections of the brain, and stroke, which often lead to epilepsy [8,9]. Thus, it is crucial to diagnose epilepsy correctly and to provide the correct treatment to patients. The problem of detecting epileptic seizures by EEG can be resolved by deep analysis of EEG epileptic signals investigating non-linear and linear features through analysing their features using innovative classification techniques to obtain an efficient detection rate [10,11,12,13,14]. In this paper, we develop an expert model to analyse epileptic EEG signals and obtain an excellent recognition rate.

Seizure activities are usually detected visually by inspecting EEG recordings. This work requires significant expertise, time, and effort [15]. Moreover, the results of this method can depend on the level of experience and expertise of individual medical professionals. Experts depend on different techniques to capture brain activity and detect seizures, such as electroencephalograms (EEG) and magnetic resonance imaging (MRI) [16,17]. However, researchers are in favour of EEG for epilepsy diagnosis due to it is low cost; it also provides supportive proof of seizures and assists with detection of epilepsy [18,19,20,21]. In addition, clinical studies have shown that a seizure can leave signs on a patient’s EEG recording even after it occurs. However, in most cases it is quite difficult to classify epileptics using EEG signals manually. Hence, developing an automated epileptic classification model can be considered an indispensable medical diagnostic to support doctors in carefully analysing EEGs. 

The problem of automatic epileptic seizure recognition has been discussed for many years. As a result, different approaches have been utilised to detect EEG seizures. Theoretically, these approaches are based on machine learning algorithms and involve several steps, starting with data analysis, then extracting features and selecting features to predict seizures. One of the earliest examples was an automatic technique proposed by Gotman [5] to detect seizures depending on the rhythm of an EEG. Another study by Theodore et al. [22] used 1BF-fluorodeoxyglucose with positron emission tomography to study clinical absence and generalized seizures. Senhadji and Wendling [23] recommended wavelet transforms and time-frequency algorithms to investigate EEG (ictal and inter-ictal) signals. Nigam and Graupe [24] applied a multistage nonlinear pre-processing filter based on an artificial neural network approach. Kannathal et al. [25] evaluated several entropy estimators to designate normal data from epileptic EEGs. Acharya et al. [26] suggested a new technique based on recurrence plots for automated identification of epileptic EEG data. 

More recently, research has focused on using convolutional neural networks (CNNs), principal component analysis (PCA), generalized linear models (GLM), global volatility index (GVIX), Tunable-Q wavelet transforms (TQWT), neural network models (NNM), fractal dimensions (FD), Recurrence Quantification Analysis (RQA), Cross-frequency coupling (CFC), and Discrete Wavelet Transforms (DWT), as discussed in the following [26,27,28,29,30,31]. Lu and Triesch [14] proposed a CNN model with residual connections to detect seizures from raw EEG data. An automatic epileptic EEG detection method based on CNN with two innovative improvements in a data classification problem was proposed by Wei et al. [32]. Türk and Özerdem [33] adopted CNN to demonstrate its ability to learn the properties of scalogram-based images. Hu et al. [34] combined CNN with an SVM for epileptic state detection. Capitán et al. [26] used PCA and distribution of power in different frequency bands to detect epileptic seizures accurately. Miao et al. [35] suggested GVIX to measure holistic signal fluctuations in wavelet coefficients and the original time-series signal. A TQWT method was applied by Bhattacharyya et al. [36] to detect epileptic seizures. San-Segundo et al. [37] used a deep neural network model to analyse epileptic EEG signals. Gruszczyńska et al. [6] applied Recurrence Quantification Analysis to classify epileptic EEG signals, and Yu et al. [28] investigated frequency bands during an epileptic event in a given patient using cross-frequency coupling. Tzimourta et al. [38] used DWT to identify epileptic EEG segments. Table 1 presents a summary of these previous studies on seizure detection. 

Despite significant efforts made by researchers in developing seizure detection models, the Federal and Drug Administration has not yet formally approved an artificial intelligence approach or a health informatics system for analysing epilepsy EEG signals. An efficient and effective artificial intelligence-based epileptic EEG classification model requires not only high accuracy but also good stability for different patients and a high-speed classification rate. Hence, in this paper a new automatic seizure detection system for epileptic EEG detection is proposed. We used two publicly available EEG datasets, the Bonn University intracranial EEG dataset and the Bern–Barcelona focal and non-focal dataset [39,40], to evaluate the proposed model. The presented model outperforms all previous machine learning algorithms. In this study, a determinant of covariance matrix (Cov–Det) coupled with an AdaBoost Back-Propagation neural network (AB_BP_NN) is used for seizure detection. The major contributions of this research are listed as:

1. A Cov–Det model is proposed to reduce the dimensionality of EEG data and explore the effective features set to detect EEG seizures. This model aims at reducing the complexity of the process, and returns results in less time.
2. The AB_BP_NN is designed and used to classify EEG features into seizure and seizure-free data. The developed model is a new and innovative classifier.
3. To eliminate redundant features, the Kolmogorov–Smirnov (KST) and Mann–Whitney U (MWUT) Tests are integrated, by which the extracted features are investigated and ranked based on KST and MWUT metrics and arithmetic operators adopted to deduce the most pertinent classified features for each pair of the EEG signal group.

**Table 1:** A summary of recent seizure-detection methods.

Authors	Methods	Cases
Nicolaou and Georgiou [41]	Permutation Entropy	A, B, C, D, and E
Srinivasan et al. [42]	Approximate entropy	A, B, C, D, and E
Lee et al. [11]	Wavelet transform, phase-space reconstruction and Euclidean distance	A, B, C, D, and E
Ahmedt-Aristizabal et al. [43]	End-to-end Training Scheme	A, B, C, D, and E
Lu and Triesch [14]	Modern Deep Learning Methods	A, B, C, D, and E
Siuly et al. [44]	Hermite Transform	A and E
Kabir and Zhang [45]	Optimum allocation technique	Two sets *A* and *E*
Tawfik et al. [46]	Weighted permutation entropy blended	A, B, C, D, and E
Şengür et al. [47]	Local Binary Pattern based approach	A and E
GulerandUbeyli et al. [7]	Wavelet Transform, Lyapunov Exponents	A, B, C, D, and E
Khan and Farooq [8]	Wavelet Transform	A and E
Ahammad et al. [48]	Discrete Wavelet Transform	A, D and E
Tzallas et al. [49]	Time-Frequency	A and E
Das et al. [50]	Dual Tree Complex	A, D and E
Liang et al. [12]	Principle component analysis, and genetic algorithms	A, D and E
Nigam and Graupe [51]	Nonlinear pre-processing filter	A and E
Polat and Güneş [52]	Fast Fourier transform, Decision Tree	A and E
Kannathal et al. [24]	Entropy Measures	A and E
Ghosh-Dastidar et al. [4]	Chaos theory and wavelet analysis, PCA	A, D and E
Tzallas et al. [53]	Time-Frequency Analysis	A, B, C, D, and E
Madhu et al. [54]	Time domain methods, frequency domain methods, and time frequency methods	A, B, C, D, and E
Patidar and Panigrahi [28]	Entropy based Tunable-Q wavelet	A and E
Subasi et al. [55]	genetic algorithm and particle swarm optimization	A, B, C, D, and E

Classes A and B were collected from five healthy subjects; classes C, D and E were recorded from EEG recordings of five epileptic patients.

## 2. Materials and Methods

**Figure 1:**
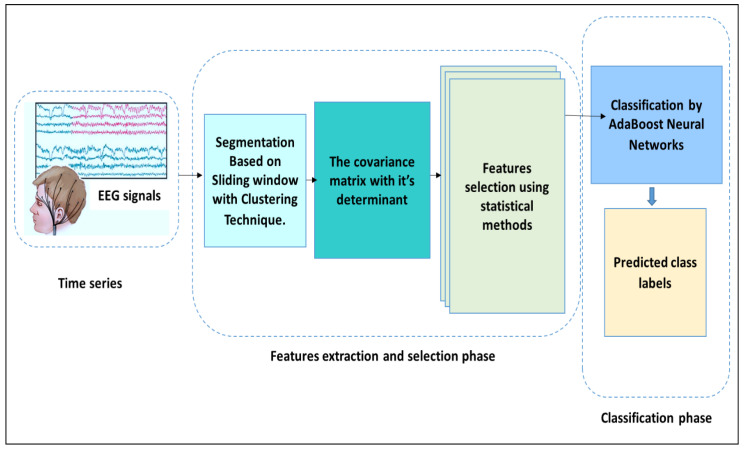
The proposed methodology for EEG signal analysis.

In this research, a new framework is proposed utilising the AdaBoost Back-Propagation neural network (AB_BP_NN) coupled to a determinant of covariance matrix (Cov–Det). To implement this model, first, each EEG signal was divided into small epochs, and furthermore, each epoch was split into sub-segments. Then, the Cov–Det model was applied to each EEG sub-segment to reduce the dimensionality. A set of statistical features, denoted as the standard deviation, variation, skewness, median, maximum, minimum, mean, mode, range, and kurtosis were pulled from each EEG sub-segment. To eradicate the redundant features, the extracted features were then investigated using two statistical metrics based on arithmetic operators, namely, the Kolmogorov–Smirnov and Mann–Whitney U Tests. To classify the selected features into normal and abnormal EEG segments, the hybrid AB_BP_NN was designed. Figure 1 shows the general methodology of the proposed model tested for the classification of epileptic EEG signal

### 2.1. Segmentation

In this study, we adopted our previous study to segment EEG signals [18,19,20,21]. Evidently, the proposed method granted a highly satisfactory classification accuracy. Mathematically, this process is explained as follows: let an EEG signal be denoted as $X=\left\{x_{1},\;x_{2},\;\ldots ,\;x_{n}\right\}$ where n is the data point. In this study, the EEG signal X is segmented into n segments, with each of those segments divided into m intervals. Each segment is divided into 32 sub-segments to extract the statistical features (Diykh, Abdulla, et al., 2019). During the training session, the number of sub-segments is empirically selected. The redundant data in each sub-segment are eliminated by extracting a set of statistical features. Consequently, each EEG epoch is denoted by a one-dimensional matrix of (f ∗ m) characteristics where f refers to the number of statistical characteristics and m is the number of sub-segments. For example, the epileptic EEG data contained five groups, A–E, with each group having 100 single channels containing 4097 data points. Each single channel was divided into four segments (1024, 1024, 1024 and 1025), then each interval was divided into 32 sub-segments.

### 2.2. Features Extraction

EEG signals are non-stationary with no-specific patterns. In this paper, we designed the Cov–Det model to reduce the dimensionality of EEG signals and extract the most powerful characteristics. 

#### 2.2.1. Covariance Matrix

Each pair of elements at the position i, j in a covariance matrix is defined as the covariance between the *i*th and *j*th elements of a random vector [56,57]. By using the covariance, the entries of the covariance matrix could be calculated for a random vector A_ij=σ(a_i,a_j ) where A∈R^(n×n) with mean vector m represents the dimension or number of random variables of the data (e.g., the number of features). In addition, the covariance matrix is symmetrical because σ(a_i,a_j )=σ(a_j,a_i ). With respect to the properties of the covariance matrix, the diagonal entries are the variances and the other entries are their covariances [58]. Accordingly, a covariance matrix is sometimes called a variance–covariance matrix [59]. The important properties of covariance matrices were summarised in our explanation of the same scenario in our earlier study [60].

#### 2.2.2. Determinant

The determinant of a matrix is a number (scalar) gained from the elements of a matrix by specified operations, and is an attribute [60,61,62]. The determinants are defined for square matrices only [63]. A determinant is denoted by (Det), or by | | for a square matrix. For a determinant in which each element in any row, or column, consists of two terms, the determinant can be expressed as the sum of two other determinants.

#### 2.2.3. Determinants of Covariance Matrix Determinants (Cov–Det)

Based on basic linear algebra, the determinant can capture how linear transformation changes area or volume and changes variables in integrals. This leads to a process of eliminating the repetition and similarity in computing the high dimensionality of the database, which was our main target behind the integration of these two approaches, that is, covariance matrix and determinant. 

In this study, the matrix elements of EEG time series with each point having its own characteristic (e.g., time index, magnitude, slope, distance to mean, etc.) contained fundamental information that could potentially be used in the present disease classification problem. The primary reason for the utilization of Cov–Det as a data shrinking method was to reduce the dimensionality of EEG signals. Initially, a time series can be described as a sequential combination of F points or written more formally as a vector of length$\;F\left(\left[x_{1},\;\ldots ,x_{F}\right]\right)$. The feature candidates can therefore be combined in a feature vector set for a point in the EEG time series. Let $\left\{v_{i}\right\}$ be the number of features defined for a point, K. The feature vector for the Nth point of the subsequence is

(1) $$a_{n}=\left[v_{N1},\;\ldots ,v_{Nk}\right];$$

when feature vectors are merged for all points, this leads to a feature matrix *A*,

(2) $$A=\left[\begin{array}{ccc} v_{11} & \cdots & v_{1k}\\ \vdots & \ddots & \vdots \\ v_{M1} & \ldots & v_{Mk} \end{array}\right].$$

The covariance of the feature matrix is

(3) $$(H_{A})=\frac{1}{F-1}\overset{F-1}{\underset{i=1}{\sum }}\left(A_{i}-m\right){\left(A_{i}-m\right)}^{T}$$

where μ is the mean vector of feature vectors $\left\{a_{1},\;\ldots ,a_{M}\right\}$. 

To improve the extraction process, this study aimed to compute the determinant of covariance matrix. Based on the essential properties of this covariance matrix, the $H_{A}$ can be symmetric (i.e., self-adjoint) with the usual inner output its eigenvalues, which are all real and positive, and the eigenvectors that belong to distinct eigenvalues orthogonal:

(4) $$H_{A}=\vee \wedge \vee^{T}.$$

Consequently, the determinant of the $H_{A}$ is

(5) $$\left|H_{A}\right|=\left|\;\vee \wedge \vee^{T}\right|=\left|\;\vee \right|\left|\;\wedge \right|\left|\;\vee^{T}\right|=\left|\;\wedge \right|\left|\;\vee \right|\left|\;\vee^{T}\right|=\left|\;\wedge \right|\left|\;\vee^{T}\vee \right|=\left|\;\wedge \right|\left|I\right|=\overset{F}{\underset{i=1}{\prod }}\gamma_{i}\;$$

In this paper, the EEG signals were represented as a one-dimension matrix. Initially, EEG time series were re-arranged to create a square matrix based on covariance matrix. The total number in the square matrix refers to the segmentation length. The Cov–Det was applied to each row; as results, we obtained a vector of 32 × 10, where 10 denotes the characteristics extracted. Hence, the dimensionality of each segment was reduced from 1024 datapoints to 320 data points. The dimension of each single EEG channel was decreased from 4097 datapoints to 1280 data points. 

### 2.3. Features Selection Based on KST and MWUT

#### 2.3.1. Stage One: Kolmogorov–Smirnov Test (KST)

The Kolmogorov–Smirnov test (KST) is a widely-used nonparametric method for testing the equivalence of continuous or discontinuous groups by utilising one-dimensional probability distributions to compare a sample with a reference probability distribution (i.e., one-sample KST) or comparing two samples (i.e., two-sample KST) [63]. A two-sample KST test is a useful nonparametric method for comparing two groups, as it is sensitive to differences in both the location and the shape of the empirical cumulative distribution functions of the two samples [3,13]. 

#### 2.3.2. Stage Two: The Mann–Whitney U Test (MWUT)

The Mann–Whitney U test (MWUT) is referred as the Mann–Whitney Wilcoxon Test or the Wilcoxon Rank Sum Test. It is applied to test whether two samples are derived from the same population [64,65]. This test is carried out as a two-sided test and thus the research hypothesis indicates that the populations are not equal, as opposed to specifying directionality [66,67].

#### 2.3.3. Two-Stage Features Selection Method

**Figure 2:**
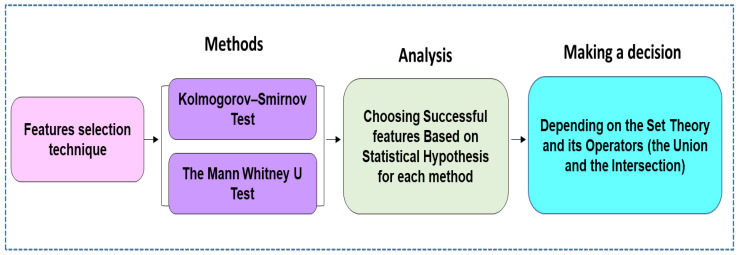
Two-stage feature selection method. Note: Stage 1 was obtained the by Kolmogorov–Simonov and Stage 2 by the Mann–Whitney U test.

The idea behind the feature selection process is to remove redundant features. By removing irrelevant data, this ensures that a classification model is trained only on the most important features [66,67,68]. In addition, removing irrelevant information can be expected to increase the accuracy of a predictive model [69] and reduce the computation time involved. Based on statistics applied to measure the similarity and dissimilarity of the means of two independent samples, this study also employed a nonparametric test that was deemed appropriate for comparing two independent samples. Generally, to compare the outcomes between independent samples, there are two popular nonparametric tests, the Kolmogorov–Smirnov test (KST) and the Mann–Whitney U test (MWUT). These two non-parametric tests were employed to reduce the dimensionality of input features fed to the classifier algorithm. Figure 2 shows the process of obtaining EEG features according to the statistical theory by which the most distinguishing features were extracted from the EEG dataset. 

Table 2 and Table 3 report the feature sets that successfully passed the two non-parametric tests. It is noteworthy that compound events in each EEG signal can be captured from diverse sample points. The set theory and its operators, with the most basic operators being the union and the intersection of the EEG features, can describe these operations. Based on the set theory and its operators, the features selected were those where each event was categorized using a diverse set of characteristics. Table 4 reports the selected features set for each event according to our investigation in Table 2 and Table 3. For example, to categorize the class {A, B and C} against class E, thee features sets were investigated (C vs. E., A vs. E and B vs. E) in order to obtain a superior representative feature dataset.

In Table 2, it can be seen that not all of the EEG groups to have the same features. This depends on the hypothesis of the test $H_{0}:\;$two samples have the same continuous distribution vs. $H_{1}:$ two samples do not come from the same continuous distribution with a level of significance α = 0.05. Based on the KST metric, the features [min, Mode, range, var, standard deviation, and max] were used to distinguish between groups B vs. E. However, to distinguish between class C vs. class E, the features set [standard deviation, kurtosis, max, min, Mode, range, and var] were accepted with the values ≤0.05, while the values ˃0.05 were rejected. At the second stage, a further investigation was made for the input features using the KST. The results in Table 3 were obtained using MWUT test to select the most appropriate features. The same hypothesis was considered with the MWUT test; for example, all feature values ≤0.05 were accepted, while those exceeding this threshold were marked as not significant with green shading. 

### 2.4. AdaBoost Back-Propagation Neural Network (AB_BP_NN)

This study develops the AB_BP_NN method based on successful implementation of a back-propagation neural network in an EEG classification problem for abnormal event detection [70]. To enhance the performance of traditional neural network models, the AdaBoost technique resulting in the hybrid AB_BP_NN was proposed, as the AdaBoost neural network is less vulnerable to issues of data over-fitting compared to some of the other machine-learning algorithms. To resolve this problem, in this study about 15% of the data from the training set were subsequently used to validate each of the neural networks. Figure 2 shows the architectural structure of the proposed hybrid AB_BP_NN model. The procedure of implementing AdaBoost Back-Propagation neural network model was as follows.

Let N be a set of the weak classifiers. This study trained the *i*th neural network on the $x_{i}\;\mathrm{and}\;y_{i}\;$sets and then evaluated the classification output of the testing set$\;y_{i}^{class}$, where the distribution D was used to calculate the evaluation error for the *i*th neural network defined as

(6) $$D_{i+1,j}=D_{i,j}X\left(1+\delta *I\left(y_{j}-y_{i,j}^{class}\right)\right)with\;\{\begin{array}{c} i=1,\ldots ,L\\ j=1,\ldots ,M \end{array}\;$$

Here, δ is multiplication factor, and $D_{i,j}$ is the *i*th in D vector. The *i*th neural network assessment error E with the equivalent distribution error D is

(7) $$E=\sum_{j=1}^{M}|D_{i,j}XI\left(y_{j}-y_{i,j}^{class}\right)|$$

Here, I is a binary function:

(8) $$I\left(x\right)=\{\begin{array}{c} 1\;if\;x>0.2\\ 0\;otherwise \end{array}$$

A weight w is assigned for the *i*th neural network based on its error, E. Then, the *i*th neural network classifies p based on the input, f. For each neural network, the weights and biases are initialized and the error threshold for I is set to 0.2. To convert the error of each neural network into its respective weight and to provide each neural network with low error and high weight, a covert function is utilised so that w for each neural network is

(9) $$w_{i}=\frac{1}{E_{i}}\;$$

Here, $w_{i}$ is the weight of *i*th neural network. The overall classification score is given by the weighted sum

(10) $$Q=\overset{m}{\underset{i=1}{\sum }}w_{i}Xp\;$$

**Figure 3:**
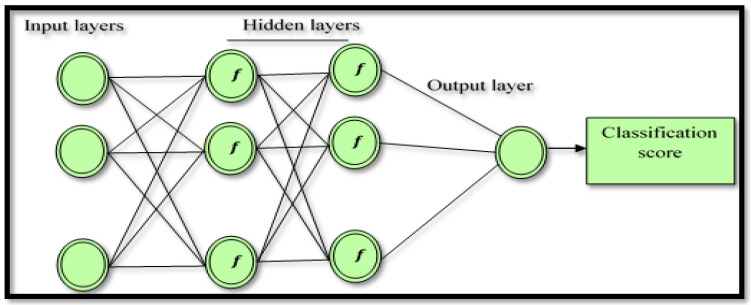
The structure of the newly proposed AdaBoost Back-Propagation neural network (AB_BP_NN) model applied for EEG signal classification purposes and subsequent epileptic disease identification.

The classification score is bounded by [0, 1], with a better score being close to a trivial value. The AdaBoost neural network was employed to classify the FC and the NFC EEG signal, with the input of the AdaBoost neural network being the extracted features in the EEG signal. In this study, a total of nine input cells were applied based on the number of the input features: two hidden layers with nine cells each. As used in most deep learning algorithms, two transfer functions denoted by the tangent sigmoid (tansig) and the rectified linear unit (ReLU) function were selected for the first and the second hidden layer, respectively, whereas a pure linear transfer function (*x*) = *x* was used for the single node output layer. In the hidden layers, several tests were performed using various activation functions, with tansig and ReLU used to select the best performance. Figure 3 shows the proposed AB_BP_NN model.

### 2.5. Performance Evaluation Metrics

To test the performance of the proposed AB–BP–NN model, several metrics were employed: accuracy (ACC), sensitivity (Sen), specificity (Spec), Negative Predictive value (NPV), f-scor (FSCOR), informedness (INFO), negative likelihood ratio (NLR), false negative rate (FNR), positive likelihood ratio (PLR), diagnostic odds ratio (DOR), false positive rate (FPR), and Mathews correlation coefficients (MCC) (Altman & Bland, 1994), (Baldi et al., 2000), (Youden, 1950). Based on the confusion matrix, the metrics of terminologies based on true positives (TP), false negatives (FN), true negatives (TN) and false positives (FP) were also calculated. Table 5 shows a short description of the score metrics used for the performance evaluation [71,72,73,74,75,76].

## 3. Results

To evaluate the proposed model, two different EEG datasets collected from Bern–Barcelona and Born University were used to detect EEG seizures. The FC and the NFC EEG datasets included a sufficiently long EEG series of 3750 pairs of FC and NFC EEG signals, while the epileptic EEG dataset contained five groups named as A–E, with each group recorded from 100 single channels. 

MATLAB R2019 was utilised to implement the proposed model. Signal processing toolbox was used in the implementation. In addition, EEG recordings and annotation files were read and stored on a PC using a MATLAB code named Read- Data_EDF collected from MathWorks.

### 3.1. The EEG Datasets 

#### 3.1.1. The Epileptic EEG Database

The epileptic EEG signal database collected from the Department of Epileptology at the University of Bonn, Germany (accessed 8 July 2021, available online https://www.ukbonn.de/epileptologie/?idcat=193&lang=3 ) is adopted in this paper [10]. This database consists of five feature sets, denoted A–E, with each feature set containing 100 channels running a length of 23.6 s from the five separate classes. Each signal was chosen based on visual inspection for artefacts, such as the cause of muscle activities or eye movements. With the same 128-channel amplifier system, all EEG recordings were made utilising an average common reference. Utilising the 12-bit resolution, all the recorded datasets were digitised at 173.61 samples per second. The 10–20 system of electrode placement was used for the recording of the EEG signals [10].

#### 3.1.2. Focal and Non-Focal Database

Another dataset, the Bern–Barcelona dataset collected from the University of Bern Department of Neurology [38,39], was employed for performance evaluation. A total of 3750 pairs of focal (FC) and non-focal (NFC) signals were collected from five patients. Pharmaco-resistant temporal lobe epilepsy was involved in the recording of EEG signals. The EEG signals were labelled as X and Y for FC and NFC. Depending on the respective channel and visual identification by two neurologists, the FC recordings from all five subjects were captured. These recordings were utilised to distinguish the first ictal EEG change. However, NFC signals were collected from channels within the neighbourhood of FC channels. All of the other channels were categorised as FC EEG channels. The sampling frequency of all EEG recordings was kept at 512 Hz, and each contained 10,240 samples. This research aimed to evaluate the proposed approach utilising all FC and NFC signals. 

### 3.2. Case 1: Classification Results for Epileptic EEG Data 

In this section, the proposed model was assessed using epileptic EEG data. Eight experiments were conducted to obtain a clear picture of the efficiency of the proposed model. In each experiment, different pairs of EEG cases were considered as follows.

⇒ Exp.1: {A vs. E}
⇒ Exp.2: {B vs. E}
⇒ Exp.3: {C vs. E}
⇒ Exp.4: {D vs. E}
⇒ Exp.5: {(A, B) vs. E}
⇒ Exp.6: {(C, D) vs. E}
⇒ Exp.7: {(A, C, D) vs. E}
⇒ Exp.8 {(A, B, C, D) vs. E}

The EEG data were divided into two equal groups for training and testing, respectively. Table 6 shows the performance of the proposed model for different EEG cases. The features in Table 4 were considered for each pair of EEG cases. Twelve metrics were used to evaluate the performance of the model with classification accuracies of eight cases. The average of classification accuracy of the proposed model was 98%, with an average sensitivity and specificity of 99% and 98%, respectively. In addition, the proposed model also gained high scores for the other performance metrics, as showed in Table 6. 

**Figure 4:**
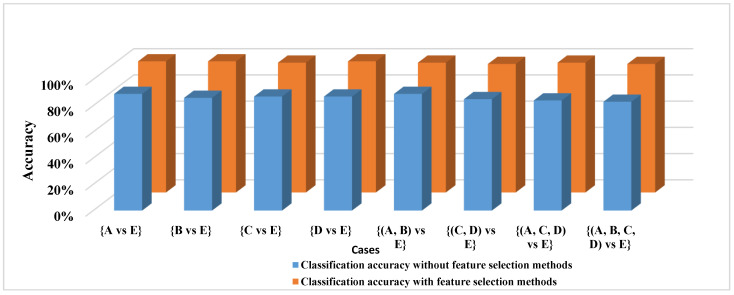
The classification accuracy of the proposed model with and without feature selection.

To further investigate the findings in Table 6, all features, including [Mean, max, min, mode, median, range, variance, standard division, Skewness and kurtosis], were adopted to classify all of the EEG cases, and these features were sent into the proposed AB_BP_NN without the feature selection phase. The results demonstrate that using the same features set to classify all EEG cases can degrade the classification accuracy. Table 7 and Figure 4 reports the classification accuracy of the proposed AB_BP_NN model with and without the feature selection phase. As most of the epileptic EEG data are non-ictal, a new experiment that reflected the actual situation of EEG data was designed to test the proposed model. In this experiment, the epileptic EEG signals were separated into two different sets.

The first set comprised all ictal EEG data while the second set represented the 25% of the non-ictal EEG data in the four non-ictal sets A–D. The experiment was repeated several times, with each 25% of the non-ictal sets A–D considered. Based on the results, the proposed model attained a satisfactory performance in all of the experiments, with an average accuracy of 97%. Table 8 shows the performance of the proposed model through a ten-cross-validation process for each EEG case. An overall classification accuracy of 99% was obtained. From the results in Table 8, it can be observed that the classification accuracy was satisfactory, and was able to reflect the efficiency of the proposed model. In addition, the performance of the proposed model was stable and there were no high fluctuations in the obtained results among the ten crosses.

### 3.3. Case 2: Classification Results for the FC and NFC EEG Data 

This section discusses the classification results of the proposed model for the FC and the NFC EEG signal. The same scenario as for the epileptic EEG data was applied to segment the FC and the NFC EEG signal and to extract the most influential features in the EEG signal. Table 9 reports the performance of the proposed model based on the sensitivity, specificity and classification accuracy against other classification models, including k-means, LS-SVM, KNN and multi-class SVM, and neural networks. Evidently, the classification accuracy of the proposed model for all subjects was higher than the k-mean, LS-SVM and multi-class SVM and neural network models. The average sensitivity and specificity of the proposed model was 98.7% and 99.37%, respectively, while the LS-SVM scored the second-highest classification accuracy with 90%, showing the efficacy of the Cov–Det-based AB_BP_NN model. 

**Figure 5:**
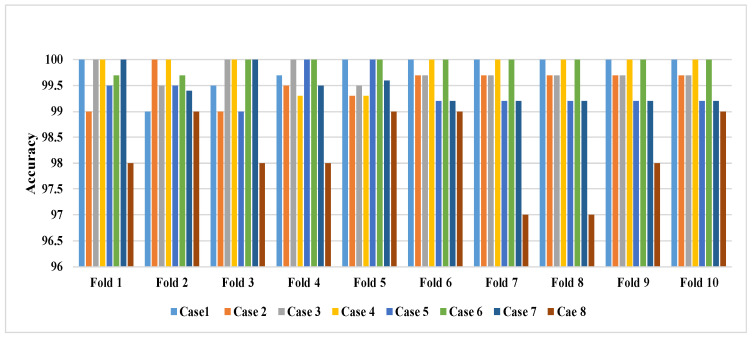
The performance of the proposed Cov–Det-based AB–BP–NN model using the ten-cross validation procedure.

To further explore the utility of the proposed model, another experiment was conducted using the ten-cross-validation procedure. Based on the results in Figure 5, the performance of the proposed method was relatively stable, and there were no high variations in the attained results among any of the ten-fold cross validations. 

## 4. Discussion

In this study, we detected epileptic seizures with a high success rate. Specifically, we used EEG data from two datasets to investigate the ability of the proposed model in seizure detection. We highlight our findings according to the following points.

**Figure 6:**
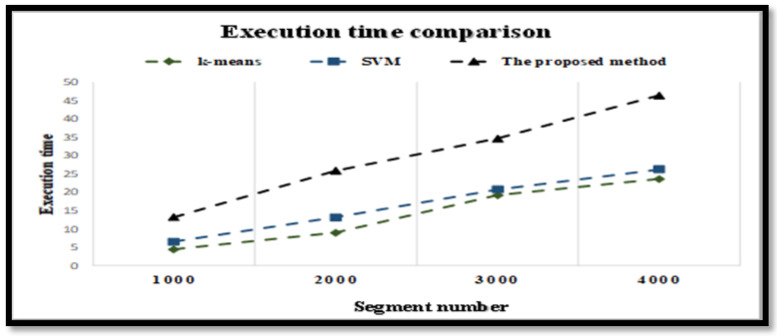
The complexity time of the proposed model for different numbers of samples with FC and NFC EEG signals.

1. The complexity of the proposed model was investigated. Figure 6 depicts the time in seconds of the proposed model based on the number of samples. For the FC and NFC EEG signals, the x-axis refers to the number of samples and the y-axis denotes the complexity time. From Figure 6, it can be noticed that the proposed model had a slightly higher execution time than the SVM and k-means methods. However, the increase in the execution time is reasonable compared with the increase in classification accuracy.
2. One of the limitations of the proposed method is that it should be tested with larger clinical databases. We believe that the proposed method may or may not yield perfect classification accuracy. The proposed model could be modified by testing other feature selection methods. Second, the proposed model could be computationally costly, especially when used in real time applications. Thus, future study should attempt to apply big data technology and use parallel processing techniques to reduce the time complexity of the proposed model and avoid the problem of feature numbers.
3. The proposed model was trained and tested based on leave-one-out cross validation (LOOCV) to avoid overfitting issues. In this experiment, one subject was used for testing while others were used for training. An average accuracy of 99% along with 99% for each dataset was obtained by the proposed model in this experiment.
4. To examine the advantages of the proposed model relative to other benchmark techniques, a comparison was made among the proposed model and several existing methods in the literature. Table 10 reports the comparison results. The proposed technique achieved an average accuracy of 100% and 98.86% for the two datasets, respectively, which is considered a noteworthy improvement compared to the state-of-the-art methods in conducting comparisons with 15 studies described in this section. For FC and NFC classification, Das and Bhuiyan [49] suggested empirical mode decomposition (EMD), discrete wavelet transform (DWT) with K-nearest neighbour classifier to discriminate focal and non-focal signals. The studies of Bhattacharyya et al. [17], R. Sharma et al. [29] and R. Sharma et al. [30] proposed an automatic classification technique based on LS-SVM. However, it was evident that our proposed AB_BP_NN model system outperformed their methods. Bhattacharyya et al. [35] proposed an automatic seizure classification method based on empirical wavelet transform technique (EWT) with LS-SVM classifier to classify the 50 pairs of focal and non-focal EEG signals. Deivasigamani et al. [71] obtained an equivalent rate of accuracy to our result; however, it was applied on 50 pairs of focal and non-focal EEG signals, while we tested the proposed model on 3750 pairs of focal and non-focal EEG signals, which produced more accurate and reliable outcomes. Despite the promising results of those studies for FC, and NFC classification, their classification accuracies were lower than our proposed model.

For seizure detection, Lee et al. [11], Ahmedt-Aristizabal et al. [42], Lu and Triesch [14] achieved better than 95% rates of accuracy based on several epilepsy classification techniques such as Neural Network Classifier (ANNs), Neural Network with Weighted Fuzzy Membership functions (NEWFM), Recurrent Neural Networks (RNNs) via the use of Long Short Term Memory (LSTM) networks, Deep Convolutional Neural Network Architecture, and SVM. Patidar and Panigrahi [27] proposed Kraskov Entropy-based Tunable-Q wavelet with LS-SVM for analysis of epileptic EEG signals, obtaining an average accuracy and sensitivity of 97.75% and 97%, respectively. Subasi et al. [54] proposed a genetic algorithm and particle swarm optimization with SVM to automatically detect an epileptic seizure, obtaining an average of accuracy 99.38%. Even though the studies described above provided advanced results, the high classification accuracy of the proposed model outperformed all of them. Comparing the studies that obtained an equivalent rate of accuracy to our result, most of the methodologies have been applied on part of datasets, while we have applied the proposed model to whole datasets and analysed eight problems, which clearly show the superiority of our proposed model. Through analysing and investigating the information presented in Table 10, the proposed model can be considered an optimal technique for these databases.

## 5. Conclusions

In this study, an efficacious automated classification model for epileptic EEG signals classification was proposed. It was evaluated using two separate medical datasets. The proposed model was evaluated by using several metrics to test its performance. Based on our findings, we believe that the proposed model can be utilised to aid neurologists and other medical specialists in the accurate diagnosis of epileptic seizures. A follow-up study may investigate the improvement of the performance of the proposed model by reducing the number of features used in this initial study. Moreover, due to the scarce number of studies focused on designing both feature extraction and detection models for the accurate diagnosis of epileptic seizures, there is a need for further research in this area.

## Figures and Tables

**Table 2:** Stage 1 of the features selection process using Kolmogorov–Smirnov metric.

Statistical Feature	A vs. E (1)	B vs. E (2)	C vs. E (3)	D vs. E (4)
Mean	3.6964 × 10^−12^	1.4660 × 10^−9^	4.2607 × 10^−13^	5.6969 × 10^−10^
Maximum	9.4812 ×10^−44^	2.9582 × 10^−32^	9.4812 × 10^−44^	2.3304 × 10^−35^
Minimum	1.2251 × 10^−44^	1.9582 × 10^−32^	2.7628 × 10^−40^	1.6754 × 10^−31^
Mode	5.6969 × 10^−10^	2.9582 × 10^−32^	9.4812 × 10^−44^	2.3304 × 10^−35^
Median	3.6951 × 10^−9^	1.2116 × 10^−7^	5.2233 × ^−8^	1.4670 × 10^−9^
Range	1.2251 × 10^−44^	5.1128 × 10^−33^	7.1865 × 10^−43^	8.6551 × 10^−34^
Variance	1.2251 × 10^−44^	5.1128 × 10^−33^	9.500 × 10^−44^	8.6551 × 10^−34^
Standard Deviation	1.5506 × 10^−45^	8.8103 × 10^−38^	1.9277 × 10^−39^	5.1128 × 10^−33^
Skewness	0.19	0.6742	0.0874	0.7410
Kurtosis	0.786	0.5521	0.3219	0.2770

**Table 3:** Stage 2 of the feature selection process using Mann–Whitney U metric.

Feature Statistics	A vs. E (1)	B vs. E (2)	C vs. E (3)	D vs. E (4)
Mean	0.14364	0.84789	0.13836	0.26889
Maximum	0.00001	0	0	0.00001
Minimum	0.00001	0	0.00001	0.00001
Mode	0	0.00001	0.00001	0
Median	0.22789	0.18177	0.39448	0.20432
Range	0	0	0.00001	0.00001
Variance	0.00001	0.00001	0	0.00001
Standard Deviation	0.00001	0.00001	0	0
Skewness	0.067418	0.79658	0.21952	0.076688
Kurtosis	0.73874	0.7871	0.0099791	0.023436

**Table 4:** The final features data set.

Problem	Features
A vs. E	[max, min, Mode, range, var. and standard deviation]
B vs. E	[max, min, Mode, range, var. and standard deviation]
C vs. E	[max, min, Mode, range, var., standard deviation and kurtosis]
D vs. E	[max, min, Mode, range, var., standard deviation and kurtosis]
{A, B vs. E}	{A vs. E} ∩{B vs. E}
{A, C vs. E}	{A vs. E}∩{C vs. E}
{A, B, C} vs. E	{A vs. E} ∪{B vs. E}∪{C vs. E}
{A, B, C, D} vs. E	{A vs. E} ∪{B vs. E}∪{C vs. E}∪{D vs. E}

**Table 5:** Summary description of performance evaluation metrics.

No.	Score Metric	Formula	No.	Metric	Formula
1	Acc.	(TP+TN)/(TP+TN+FP+FN)	7	NLR	FNR/Spec.
2	Sen.	TP/(TP+FN)	8	DOR	(TP/FN)/(FP/TN)
3	Spec.	TN/(TN+FP)	9	INFO.	Sen. + Spec. − 1
4	NPV	TN/(TN+FN)	10	FNR	1-Sen.
5	FSCOR	$2\times \frac{\mathrm{PPV}\times \mathrm{Sen}.}{\mathrm{PPV}+\mathrm{Sen}.}$	11	PLR	Sen./FPR
6	MCC.	((TP × TN) − (FP × FN))/√((TP + FP)(TP + FN)(TN + FP)(TN + FN))	12	FPR	FP/(FP + TN)

**Table 6:** Classification accuracy under feature selection.

Case	Sen	Spec	ACC	NPV	FNR	FPR	FSCOR	INFO	NLR	DOR	PLR	MCC
{A vs. E}	99%	98%	100%	97%	87%	97%	97%	99%	97%	98%	98%	97%
{B vs. E}	98%	99%	100%	98%	85%	98%	98%	98%	97%	98%	98%	99%
{C vs. E}	99%	99%	99%	99%	87%	97%	99%	97%	96%	97%	98%	99%
{D vs. E}	98%	100%	100%	99%	86%	99%	99%	99%	99%	99%	99%	100%
{(A, B) vs. E}	99%	98%	99%	97%	85%	98%	97%	97%	98%	97%	97%	97%
{(C, D) vs. E}	98%	97%	98%	98%	85%	99%	98%	96%	98%	98%	98%	98%
{(A, C, D) vs. E}	98%	99%	99%	99%	84%	98%	99%	99%	99%	99%	98%	99%
{(A, B, C, D) vs. E}	99%	98%	98%	98%	86%	98%	97%	97%	98%	97%	98%	97%

**Table 7:** Classification accuracy without feature selection.

Case	Sen	Spec	ACC	NPV	FNR	FPR	FSCOR	INFO	NLR	DOR	PLR	MCC
{A vs. E}	88%	87%	89%	83%	82%	81%	83%	81%	82%	83%	83%	85%
{B vs. E}	86%	88%	86%	82%	83%	81%	82%	85%	81%	81%	83%	84%
{C vs. E}	87%	85%	87%	81%	82%	83%	81%	84%	83%	82%	99%	83%
{D vs. E}	85%	84%	87%	80%	83%	81%	82%	83%	80%	81%	100%	83%
{(A, B) vs. E}	87%	83%	89%	82%	83%	81%	84%	82%	83%	83%	99%	85%
{(C, D) vs. E}	88%	85%	85%	83%	82%	83%	83%	84%	81%	81%	98%	83%
{(A, C, D) vs. E}	86%	86%	84%	82%	84%	84%	82%	82%	83%	83%	82%	82%
{(A, B, C, D) vs. E}	85%	84%	83%	81%	83%	82%	81%	82%	81%	81%	83%	83%

**Table 8:** Classification accuracy for each EEG Case.

EEG Cases	Accuracy Based on Ten Cross Validations
{A vs. E}	100%
{B vs. E}	100%
{C vs. E}	98.5%
{D vs. E}	99%
{(A, B) vs. E}	98%
{(C, D) vs. E}	98.2%
{(A, C, D) vs. E}	98%
{(A, B, C, D) vs. E}	98.5%

**Table 9:** Comparison of the proposed model with other classifiers.

Methods	Subject 1			Subject 2			Subject 3			Subject 4			Subject 5		
	Acc	Spec	Sen	Acc	Spec	Sen	Acc	Spec	Sen	Acc	Spec	Sen	Acc	Spec	Sen
The proposed model	99	98.4	99	98.7	98	98	99	98.4	97.9	98.6	97.8	97.6	99	97.5	97.5
k-means	86	85	83	89	88	86	87	83	85	88	87,3	86.5	90	88	87
KNN	90	89	88	87	88	86	89	87.5	88.4	89.5	87.9	87.6	91	89	88.9
LS-SVM	92	91	90	89	90	88	91	90	89	92	91	89	93	91	89
Multi-class-SVM	90	89	88	88	86	89	90	90	89	91	90	90	89	87	88

**Table 10:** Comparisons among proposed model with the state of the art.

Authors	Methods	Classifiers	Cases	Acc.	Sen.	Spe.
Das and Bhuiyan [49]	EMD, DWT	K-nearest neighbour	Entire Dataset	89.4%	-	-
Bhattacharyya et al. [17]	TQWT	LS-SVM	3750 pairs of focal and non-focal	84.67%	-	-
R. Sharma et al. [29]	DWT	LS-SVM	50 pairs of focal and non-focal	84%	84%	84%
R. Sharma et al. (2015b)	Entropy features	LS-SVM	50 pairs of focal and non-focal	87%	-	-
Deivasigamani et al. [71]	(DT-CWT)	ANFIS	50 pairs of focal and 50 non-focal	99%	98%	100%
Bhattacharyya et al. [35]	EWT	LS-SVM	50 pairs of focal and 50 non-focal	90%	88%	92%
Acharya et al. [26]	DFA, FD, LLE	LS-SVM	3750 pairs of focal and 3750 non-focal	87.93%	89.97%	85.89%
**Proposed Method**	***Cov–Det***	***AB–BP–NN* model**	**3750 pairs of focal and 3750 non-focal**	**98.86%**	**98.7%**	**99.37%**
**Epileptic EEG dataset**						
**Authors**	**Methods**	**Classifiers**	**Cases**	**Acc.**	**Sen.**	**Spe.**
Lee et al. [11]	WT, PSR, ED	NEWFM	Five sets A, B, C, D, and E	98.17%	96.33%	100%
Ahmedt-Aristizabal et al. [42]	End-to-end Training Scheme	LSTMs	Five sets A, B, C, D, and E	95.54%	91.83%	90.50%
Lu and Triesch [14]	DNT	ANT	Five sets A, B, C, D, and E	99%	96.15%	100%
Şengür et al. [46]	GLCM, TFCM, LBP	SVM	Two sets A and E	100%	100%	100%
Liang et al. [12]	PCA, GAs	BP, LISVM	Three sets A, D and E	96.83%	-	-
Madhu et al. [54]	TM, FT	PNN	Five sets A, B, C, D, and E	92.75%	72.5%	98%
Patidar and Panigrahi [27]	En	LS-SVM	Two sets *A* and *E*	97.75%	97%	-
Subasi et al. [54]	GA, PSO	SVM	Five sets *A*, *B*, *C*, *D*, and *E*	99.38%	-	-
**Proposed Method**	***Cov–Det***	***AB–BP–NN* model**	**Five sets A, B, C, D, and E with Eight cases (8 problems)**	**100%**	**99%**	**98%**

## Data Availability

Data available in a publicly accessible repository. The data presented in this study are openly available in https://www.ukbonn.de/epileptologie/?idcat=193&lang=3 (accessed on 8 July 2020).
